# Signal processing and resistive switching mechanisms of niobium oxides

**DOI:** 10.1080/14686996.2026.2713993

**Published:** 2026-08-07

**Authors:** Fei Zeng, Da Ma

**Affiliations:** a Key Laboratory of Advanced Materials (MOE), School of Materials Science and Engineering, Tsinghua University, Beijing, China; b Center for Brain Inspired Computing Research (CBICR), Tsinghua University, Beijing, China

**Keywords:** Niobium oxides, signal processing, resistive switching mechanism, neuromorphic computation

## Abstract

This paper reviews recent advances in signal processing and resistive switching mechanisms of niobium oxides. The reported signal-processing functionalities include neural spike firing, phase locking, frequency and amplitude modulation, information-flux control, and quantized encoding. The underlying resistive switching mechanism indicates that the phase composition within conductive filaments, or the local activity of niobium oxides, can be modulated and stabilized by acquiring appropriate energy from external electrical stimuli. Precise control over these two characteristics enables the design of artificial neural networks with high biological plausibility, thereby significantly enhancing computational efficiency.

## IMPACT STATEMENT

Niobium oxides have volatile resistive switching behaviors and capable of acting artificial neurons to encode various signals into structured data. They have potential for constructing artificial neural network with high biological plausibility.

## GRAPHICAL ABSTRACT

**Figure:**
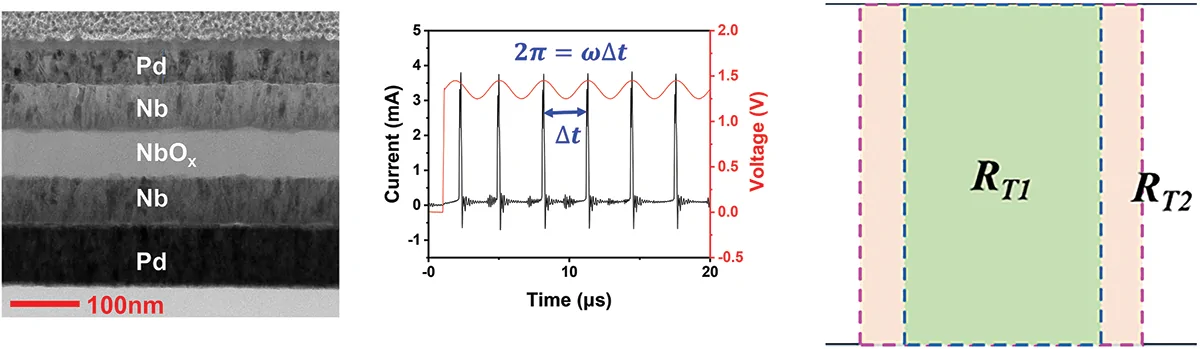

## Introduction

Over the past decade, niobium oxide devices have attracted considerable attention as candidate neurons for artificial neural networks (ANNs) [1,2]. Niobium oxides typically exhibit volatile resistive switching (RS) behavior governed by voltage/current thresholds and phase-change temperatures. Leveraging this property, researchers have developed oscillatory circuits using paired NbO_x_ devices to generate neural spike trains [3], sparking broad interest in constructing artificial networks that mimic biological neural topologies. This vision was long hindered by the need for complex circuits containing dozens of transistors to implement even basic neuron units, which greatly increased the difficulty of building compact networks [4]. Meanwhile, ANNs have evolved along a trajectory focused on the hardware implementation of machine-learning theories [5].

The first neuromorphic computing demonstration using NbO_x_-based hardware exploited chaotic oscillations to amplify random fluctuations. In this scheme, NbO_x_ devices serve as hardware accelerators for Hopfield networks, preventing the system from becoming trapped in local minima and yielding suboptimal solutions [6]. The second approach implements Boolean operations and synchronous computation using transistor-free networks [7]. The third routinely employs NbO_x_ devices as integrate-and-fire (I&F) neurons: when the accumulated signal strength (weight) exceeds the switching threshold, the device emits neural spikes [8]. This computational framework has enabled simulations of tactile, visual, and auditory functions [9]. Although these advances promote the application of NbO_x_ materials and devices, they also raise critical concerns.

Current machine-learning-based ANNs remain fundamentally von Neumann architectures, as synaptic weights must be stored in separate memory media [1,10]. Computational schemes – including algorithms and parameters – are task-specific and do not follow unified physical principles; consequently, computing pipelines must be reconfigured for new tasks, and memory modules must be rewritten when capacity is exhausted [11]. Several studies have proposed in-sensor computing to reduce data transfer between sensors and processors [12]. However, such sensor networks typically require retraining for new tasks, which contradicts the adaptability of biological sensors to arbitrary signals. Neurons behind biological sensors process inputs far more efficiently by generating structured data (featured spike trains) for subsequent neural layers [13]. Studies in neural dynamics have shown that Hodgkin–Huxley (HH) and Izhikevich neuron models – rather than the I&F model – enable sophisticated encoding and drastically improve the efficiency of spiking neural networks (SNNs) [14,15]. Fortunately, hardware implementations of these two neuron types can be realized using NbO_x_ devices, avoiding the heavy computational load of software-based emulation [7,16–18]. Accordingly, greater emphasis should be placed on generating structured data, exploring universal physical principles for logical computation, and controlling information flux. This paper briefly reviews signal processing using NbO_x_ devices and its correlation with the local activity and RS mechanisms of NbO_x_ thin films.

## Signal processing of NbO_x_ device

Once researchers recognized that responses to input signals or events can be encoded, they sought to reconstruct such signals because computation based on feature spike trains drastically reduces energy consumption [7]. A feature spike train embeds complete temporal and spatial information of a target event. Projecting these features into higher neural layers facilitates the construction of vector spaces to describe and encode complex matters. Neuroscience-oriented mathematicians have established a suite of neuron models to reproduce diverse spike patterns but have not yet identified dedicated computational functions for specific patterns [14]. SNNs built on these models consume considerable computational resources per neuron, posing severe challenges for large-scale systems. This dilemma can be alleviated or resolved using hardware neurons that eliminate complex circuits.

**Figure 1.**
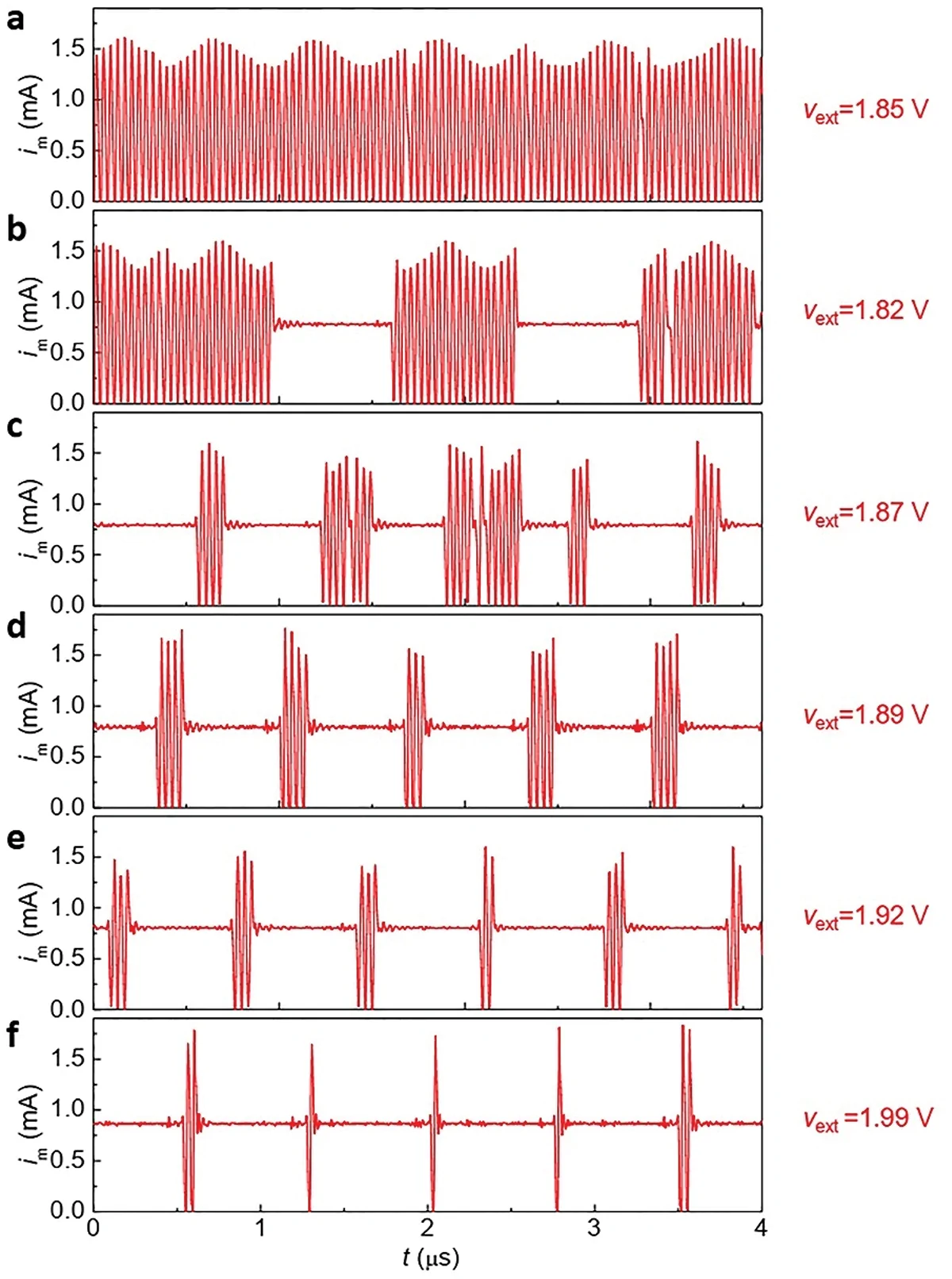
Extended experimental data on mixed-mode neuromorphic properties [7]. Temporal current through the NbO_x_ device is *i_m_*, external voltages is *v_ext_*. Self-sustained oscillations, periodic spiking, periodic bursting and burst-number adaptation coexist. (Reproduced from Kumar et al. [7] *Nature*, 2020. Reprinted with permission from Springer Nature).

Figure 1 presents experimental results emulating mixed-mode neuromorphic properties using a basic cell consisting of a NbOx device and a series resistor [7]. An external bias positions the device within the Mott phase-change region, enabling fine modulation of device states. Approximately 15 types of neural spike trains have been emulated under this operating regime. Recognition and interpretation of these spike trains enable rapid decision-making and substantial savings in computing time. Moreover, these experiments demonstrate that a single nanoelectronic device can perform third-order dynamic computations without additional overhead, which is highly favorable for large-scale integration and parallel computing.

**Figure 2.**
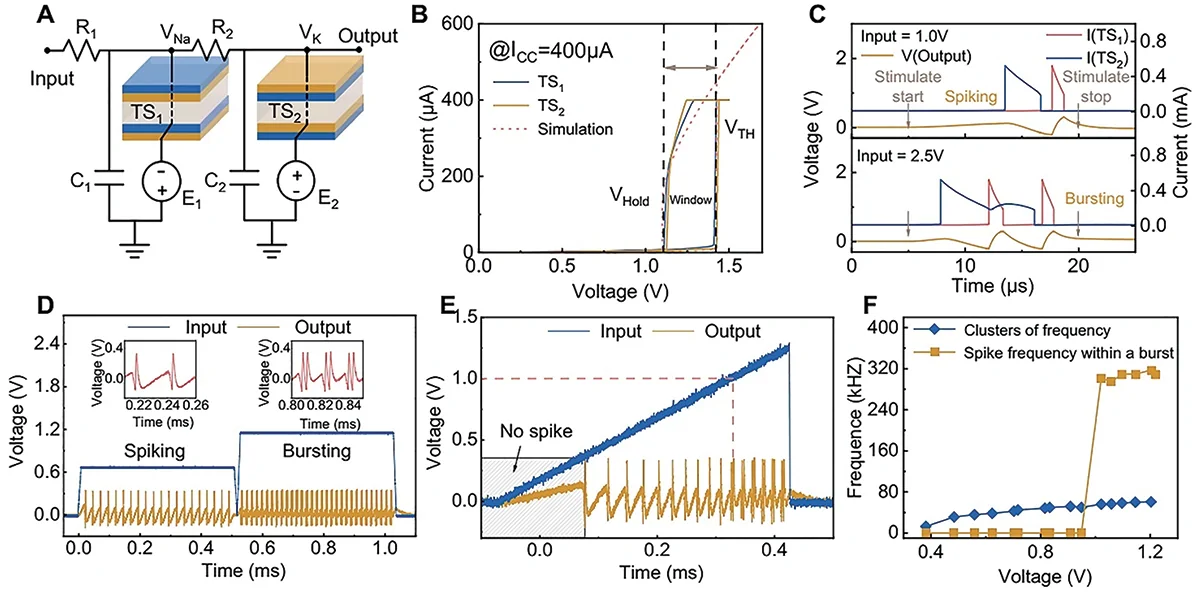
HH neuron circuit based on NbO_2_ memristors and the firing behaviors [18]. (A) The HH neuron consists of two resistors (R1, R2), two capacitors (C1, C2), and two TS devices (TS1, TS2) with opposite bias voltages provided by two voltage sources (E1, E2). (B) Typical volatile threshold switching behavior of the TS device. (C) Switching sequence of the TS devices (up panel). In a bursting feature (bottom panel), the TS2’s switching-on time is before TS1’s switching-off and TS2 switches twice. (D) The neuron fires in the spiking feature when the input is 0.7 V, and fires in the bursting feature when the input is 1.2 V. (E) The neuron shows a transition between the two firing features under triangle wave scanning with an amplitude of 1.3 V. (F) The firing cluster frequency increases linearly with the input voltage. Bursting starts to appear when the voltage input is 1.0 V. (Reproduced from Yang et al. [18] under a Creative Commons Attribution 4.0 International License https://creativecommons.org/licenses/by/4.0/).

The HH neuron aims to replicate all ionic dynamics across the neural membrane and typically comprises multiple ion channels with distinct Nernst potentials [19]. Figure 2(a) shows an HH neuron with two ion channels: a Na^+^ channel implemented using a negatively biased NbO_x_ device and a K^+^ channel using a positively biased NbO_x_ device [18]. Figure 2(d) illustrates how this HH neuron emulates two types of spike trains. At an input voltage of 0.7 V, the neuron fires spike trains in which one burst contains a single spike; at 1.2 V, a burst cluster consists of two spikes. Figure 2(e) shows the voltage-driven transition between single-spike and twin-spike firing. Emulating additional ion channels simply requires adding threshold-switching elements (*TS*_*i*_) and setting their equilibrium potentials (*E*_*i*_) to match the desired Nernst potentials, a scheme previously demonstrated using VO_2_-based *TS*_*i*_ [20].

The HH neuron renders signal control flexible and intuitive. Yang et al. demonstrated a biologically inspired selective communication circuit containing bursting-detection neurons (BDN) and spiking-detection neurons (SDN) designed with distinct circuit parameters. The resonant BDN responds only to low-frequency inputs by generating single spikes, whereas the integrative SDN responds only to high-frequency inputs by producing twin-spike bursts [18]. Nonetheless, large-scale integration of HH-neuron hardware remains challenging. Realizing Nernst potentials requires locally integrated power supplies, which is far more difficult than using a global subthreshold bias for all neurons. Future designs may eliminate the need for intrinsic built-in potentials.

**Figure 3.**
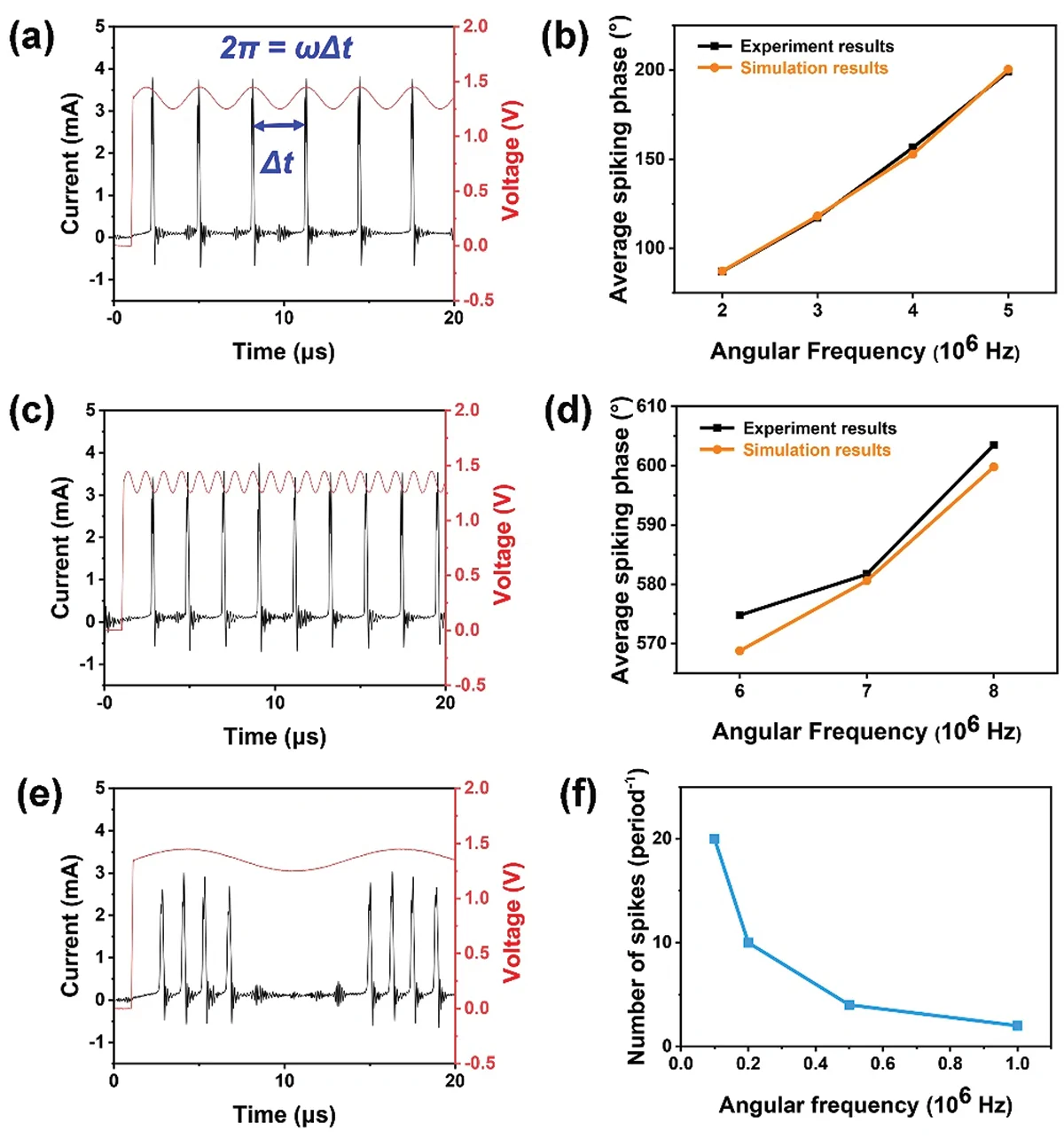
Phase locking and frequency selectivity [16]. (a) Typical current spikes (black) fire under a sinusoidal input (red) with an angular frequency of 2 × 10^6^ Hz (spikes lock at 87°). (b) Phase *vs* frequency for inputs ranging from 2 × 10^6^ to 5 × 10^6^ Hz. (c) Typical current spikes (black) fired under a sinusoidal input (red) with an angular frequency of 6 × 10^6^ Hz (spikes lock at 575°). (d) Phase *vs* frequency for inputs ranging from 6 × 10^6^ to 8 × 10^6^ Hz. (e) Typical current spikes (black) fire under a sinusoidal input (red) with an angular frequency of 0.5 × 10^6^ Hz (four spikes within a perturbation period). (f) Spike number *vs* frequency for inputs ranging from 0.1 to 1.0 × 10^6^ Hz. (Adapted from Yu, J et al. [16] under a Creative Commons Attribution 4.0 International License).

Neural spike trains contain alternating-current (AC) components, and all external signals either possess AC components or can be decomposed into sums of multiple AC components. Thus, neural systems hold inherent advantages in processing AC signals, a view supported by anatomical and functional studies of the auditory system [13]. A fundamental question arises: why do neurons maintain a resting membrane potential between intracellular and extracellular environments? The answer is that neurons are biased near the edge of chaos, enabling small signals to rapidly trigger high-order chaotic dynamics. Based on this understanding, Yu et al. proposed a novel signal-processing paradigm: they first biased a NbO_x_ device to a state slightly below its resistive switching threshold, mimicking the neuronal resting potential [19]. They then stimulated the device with a small sinusoidal signal such that the sum of the bias and signal amplitude matched the switching threshold. A key discovery was the emergence of phase locking and frequency selectivity [16]. As shown in (Figure 3(a)), the device consistently fires spikes at a phase between $\frac{\pi }{2}$ to π. The resulting spike train effectively performs a discrete Fourier transform, capturing the frequency–time relationship described by 2π=ωΔt. This relation is theoretically and practically significant, as conventional analog electronics require far more circuit resources to implement discrete Fourier transforms. Conventional phase-locked loops also rely on oscillator circuits to track and lock onto input frequencies. The physical origin of phase locking lies in the instantaneous phase change and recovery of NbO_x_, which act as a probe to extract information from the sinusoidal input.

When the input frequency exceeds 6 MHz, the phase-locking relation generalizes to 2nπ=ωΔt (shown in Figure 3(c)). The spike-firing phase scales linearly with frequency (Figures 3(b,d)). These results closely match phase-locking phenomena observed in biological hearing [13]. When the frequency drops below 1 MHz, the spike trains exhibit cluster-bursting characteristics typical of olfactory and other low-frequency sensory modalities. Spike density (number of spikes per period) generally decreases with increasing signal frequency (Figure 3(f)). Thus, the NbO_x_ device successfully acts as an Izhikevich neuron, capable of encoding multimodal signals and interfacing with broadband multisensors – an essential capability for advancing humanoid robotics.

Furthermore, phase locking underpins other key auditory functions. Yu et al. used NbO_x_ devices to emulate all core auditory senses, including intensity coding, tonotopy, frequency mixing, and sound localization. A sound wave reaching two ears creates a phase difference or interaural time delay. The synthesized signal at the medial superior olive generates spike trains that encode this spatiotemporal information. The team successfully obtained an interaural delay–spike density curve, enabling straightforward and accurate sound-direction judgment – far more convenient than conventional analog-electronic methods. In barn owls, auditory neurons are arranged in three columns, with the middle serving as a reference [21]. Such alignment can be readily replicated using NbO_x_ devices on a CMOS fabrication platform. We believe that other auditory phenomena, such as the Doppler effect [22], can also be encoded using the chaotic dynamics of NbO_x_.

**Figure 4.**
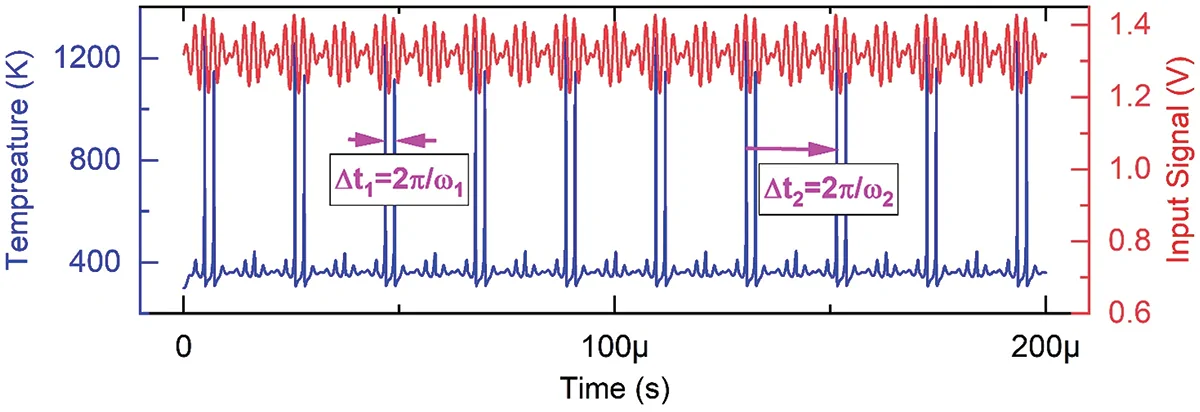
Encoding frequency modulated signals. The circuit composes of a NbO_x_ device and a 1 kΩ resistor. The responses use temperature spike train. The input is $V_{\mathrm{in}}=V_{0}+\left(V_{\omega_{1}}+V_{\omega_{2}}\times \sin\left(\omega_{2}t\right)\right)\times \sin\left(\omega_{1}t\right)$, where $V_{0}=1.32V,\;V_{\omega_{1}}=0.01V,\omega_{1}=3\mathrm{MHz},\frac{V_{\omega_{1}}}{V_{\omega_{2}}}=0.1,\frac{\omega_{1}}{\omega_{2}}=10$.

Given the high-efficiency phase locking of NbO_x_ devices, researchers are exploring their use in processing more complex signal sequences. Figure 4 demonstrates the encoding of frequency- and/or amplitude-modulated (FM/AM) signals. The input takes the form: $V_{\mathrm{in}}=V_{0}+\left(V_{\omega_{1}}+V_{\omega_{2}}\times \sin\left(\omega_{2}t\right)\right)\times \sin\left(\omega_{1}t\right)$, where $\omega_{2}<\omega_{1}$ and the switching threshold satisfies $V_{\mathrm{th}}=V_{0}+V_{\omega_{1}}+V_{\omega_{2}}$. Simulation parameters are $\frac{V_{\omega_{1}}}{V_{\omega_{2}}}=0.1\left(V_{\omega_{1}}=0.01\right)$, $\frac{\omega_{1}}{\omega_{2}}=10\left(\omega_{1}=3\mathrm{MHz}\right)$. The modulated input (red line) elicits a structured spike train (blue line) composed of periodic clusters. The inter-cluster interval is $\Delta t_{2}=\frac{2\pi }{\omega_{2}}$, and the intra-cluster spike interval is $\Delta t_{1}=\frac{2\pi }{\omega_{1}}$. Thus, the spike train encodes information for both $\omega_{1}$ and $\omega_{2}$, revealing the strong potential of NbO_x_ devices for information encryption. The responses in Figure 4 use the temperature spike train because it synchronizes with the current train through the NbO_x_ device. In addition, we can distinguish whether the NbO_x_ reaches the phase-change threshold, where a spike identifies an event occurrence.

Virtually all signal sequences – including electromagnetic and acoustic waves in communications – can be expanded via Fourier transformation. Phase locking and modulated-signal encoding give NbO_x_ devices two distinct advantages for future SNN systems. First, they do not require specialized preprocessing of raw signals. For example, rectangular waves are often used to simulate direction selectivity in vision [23], yet such waveforms rarely occur in nature. NbO_x_ devices can handle high-frequency signals using the scheme in (Figure 3(c)) or tailored bias conditions. Second, they respond over a wide frequency range [17]. Thus, a unified SNN can use identical materials, devices, physical mechanisms, and global biases to encode multimodal signals from diverse sensors [24]. This strategy drastically simplifies SNN architecture and enhances computational efficiency.

After developing novel memristors, researchers naturally investigate their cooperative behavior under proper interconnection. Several theoretical studies have been conducted, although experimental progress lags due to the lack of standardized measurement protocols. Lin et al. proposed a memristive Hopfield neural network with three coupled memristors [25]. The complex dynamics of this triple-memristor network exhibit spatially multi-structured chaotic attractors and initial-offset coexistence. These chaotic attractors can be tuned by adjusting memristor parameters, while spatially coexisting attractors are controlled by initial states, leading to extreme multistability.

**Figure 5.**
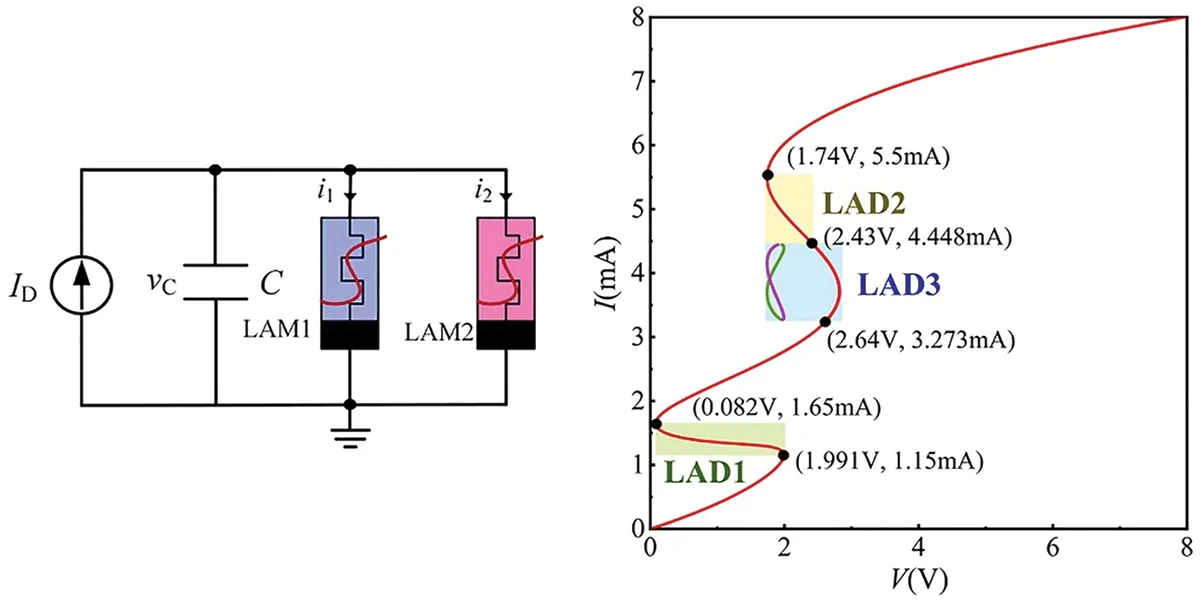
(left) Third-order chaotic neuron composed of twinned local active memristors (LAMs), a capacitor and a current source. (right) Direct-current (DC) voltage-current (V-I) curve of them [26], showing NDR regions (LAD1, LAD2) with $I\in \left\{\left[1.15\mathrm{mA},1.65\mathrm{mA}\right]\cup \left[4.48\mathrm{mA},5.5\mathrm{mA}\right]\right\}$ and multiple equilibria regions (LAD3) with $I\in \left[3.273\mathrm{mA},4.448\mathrm{mA}\right]$. (Adapted from Dong et al. [26] *International Journal of Bifurcation and Chaos*, 2025. Reprinted with permission from World Scientific Publishing Company).

Inspired by these works, researchers continue to optimize artificial neurons for advanced neuromorphic computing. Dong et al. proposed a chaotic neuron circuit integrating twin memristors and a capacitor (Figure 5) [26]. The DC current–voltage (I–V) curve exhibits two negative differential resistance (NDR) regions (LAD1, LAD2) and multiple equilibrium regions (LAD3). Modulating the input signal strength shifts the system state among these regions, triggering diverse spike patterns. The results show that two coupled artificial neurons can selectively respond to inputs, mirroring mechanisms in biological networks. As is known, biological neuron arrays differ significantly from the prevalent crossbar arrays used in artificial neural networks. For instance, the basilar membrane in the cochlea selectively responds to audio frequency along its length. However, the response may contain various vibration modes. Further filtering should occur at the following neuron arrays connected in parallel [13]. Scientists only know that neurons fire after accepting signals but do not fully understand their working mechanisms, because proper biological and physical methods to detect such selectivity or filtering are lacking. Comparing the frequency selectivity of the circuit in (Figure 5), we suppose that the vibration mode modulates the signal strength or (and) capacity in parallel to the neurons by the movement of the cochlear reticular plate. Therefore, an opportunity appears that the NbO_x_ devices to be used in constructing arrays (circuits) resembling their biological counterparts. Researchers can launch a novel field to explore the mechanisms of various biological networks by verifying the chaotic dynamics of these artificial neuron arrays.

Signals entering the neural system from sensory organs undergo multiple stages of encoding before reaching the highest cortical areas. Meanwhile, neurons in upper layers may feedback to lower layers to suppress further transmission or reroute encoded signals to other cortical regions. Thus, numerous inhibitory neurons regulate information flux [19]. For instance, barrel cortex interneurons maintain proper excitation/inhibition balance and participate in sensory processing [27]. Many studies link learning behaviors closely to cerebellar circuits [28]. Artificial neurons enable analogous investigations aligned with neuroscience frontiers.

**Figure 6.**
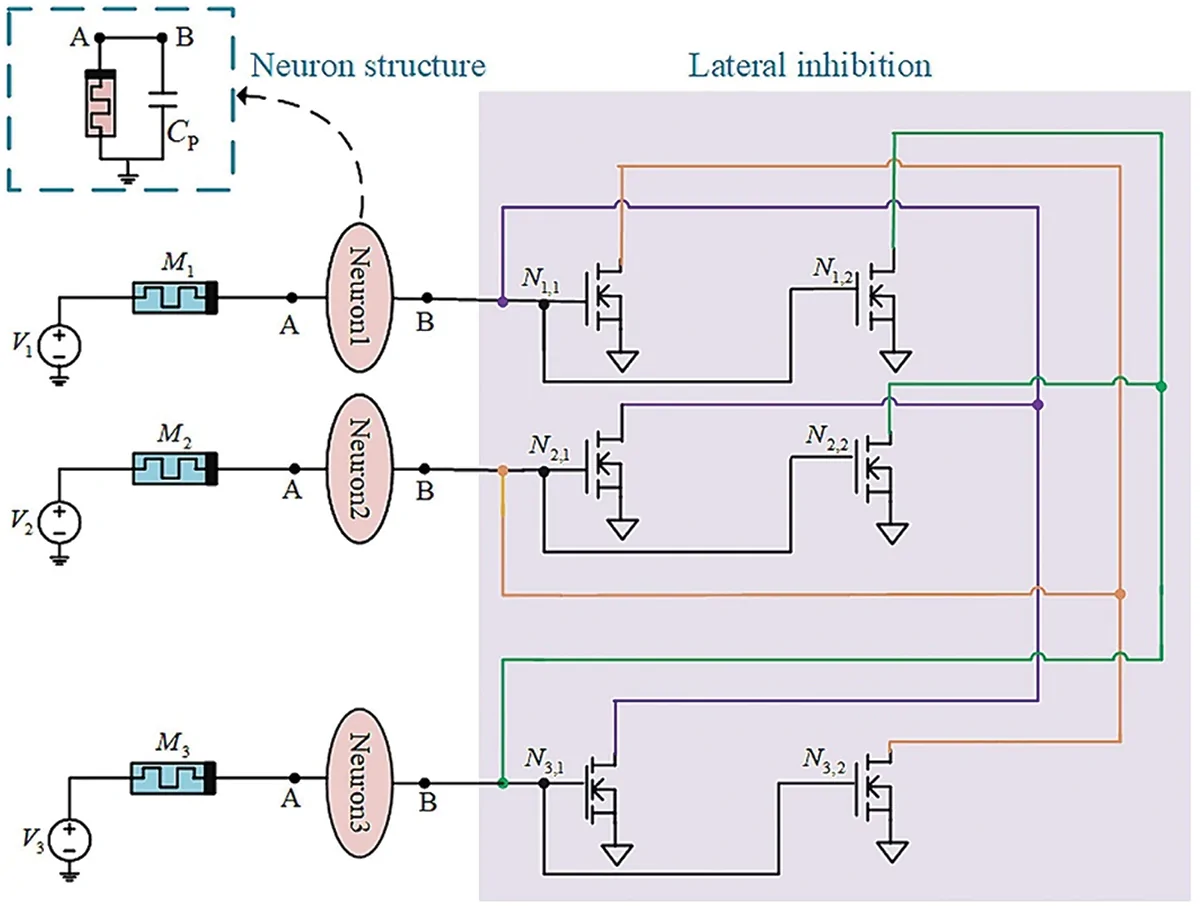
The circuit of verifying the winner strategy [30]. The neuron unit composes of a NbO_x_ and a capacitor. For a network with m output neurons, each output branch has m-1 NMOS transistors connected in parallel with the membrane capacitor of this branch output neuron. The drain terminals of all NMOS transistors are connected to the non-ground terminal of the membrane capacitor of other m-1 branch output neurons, and the gate terminals of transistors are connected to the outputs of m-1 branch output neurons, respectively. (Reproduced from Lu et al. [30] *International Journal of Circuit Theory & Applications*, 2024. Reprinted with permission from Wiley).

Competitive neural networks represent complex systems capable of controlling information flux, operating *via* excitatory–inhibitory neuron coupling [29]. Lu et al. constructed a winner‑take‑all circuit using Nb_2_O_5_ devices (Figure 6) [30]. Lateral inhibition controls NMOS transistors via their gate–source voltage. All transistor drains connect to the other *m*-1 branch output neurons, and their gates connect to the *m*-1 branch outputs. The gate threshold voltage $V_{\mathrm{GSth}}$ matches the switching threshold $V_{\mathrm{th}}$ of the output neurons. The winning neuron reaches $V_{\mathrm{th}}$ first, activating all NMOS transistors in its branch and blocking outputs from competing neurons. An inhibitory neuron turns on its corresponding NMOS transistor once the gate voltage reaches $V_{\mathrm{GSth}}$. Nb_2_O_5_ devices generate spikes with amplitudes exceeding $V_{\mathrm{GSth}}$ in the lateral inhibition module. This scheme achieves efficient winner-take-all selection and demonstrates the potential of NbO_x_ devices for information-flux control. In addition, the authors prove the practicability through a simulation on image recognition. The proposed neural network does not use any additional processor units, such as CPU or FPGA, exhibiting the advantages of low power consumption and time efficiency for neuromorphic computing.

**Figure 7.**
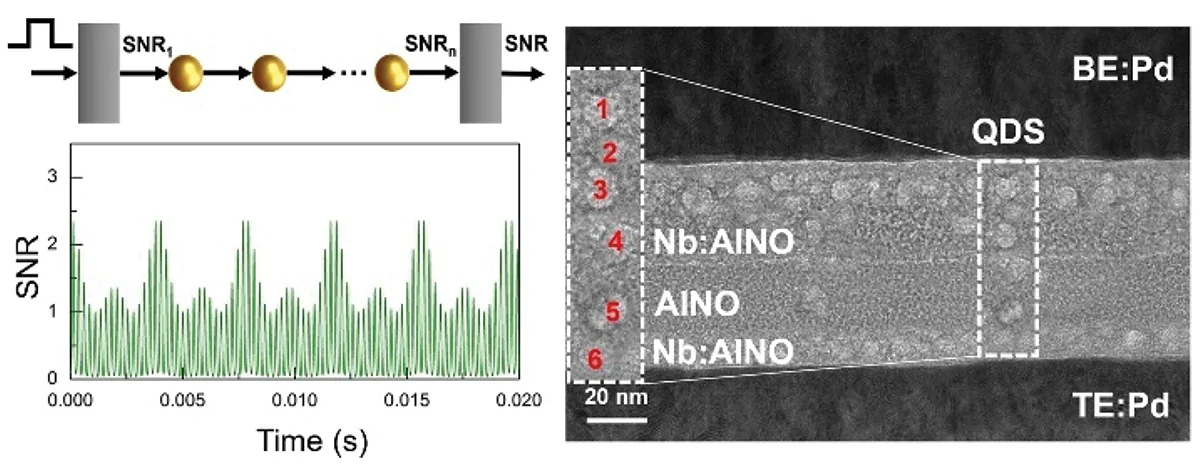
(left) Self-assembly phase-change quantum-dot string (PCQDS) responds to a long signal sequence in a modulated output style. (right) A PCQDS circled by a white dashed box is formed after the signal interaction consisted of 10,000 square pulses with amplitude of ―2 V and frequency of 30,000 Hz. An individual QD (labelled by 1–6) is a NbO nano-cluster [33]. (Reproduced from Wan et al. [33] *Nano Lett*. 2024, 24, 80898097 (Visual Abstract). Reprinted with permission from American Chemical Society).

Stochastic resonance (SR) is ubiquitous in nature and is widely used to retrieve weak signals with the assistance of noise or to generate random noise [31]. Löfstedt, et al. proposed a signal-processing protocol in which structured signals can be modulated at the output [32]. However, experimentally implementing adaptive structured noise in a system remains challenging. A recent strategy leverages the intrinsic nonlinearity of local material activity. A phase-change quantum-dot string (PCQDS) based on NbO nanoclusters was fabricated in an AlNO thin film, containing six NbO quantum dots (QDs) and seven tunnel barriers (Figure 7, right) [33]. Electron tunneling through this system requires stochastic signals to align energy levels across potential wells to satisfy coherent resonance. Tunneling indeed occurs via the SR effect. Periodic rectangular signal sequences activate the PCQDS system to produce amplitude-modulated outputs. Theoretical analysis yields the global signal-to-noise ratio (SNR):

(1) $$\mathrm{SNR}=\overset{7}{\underset{i}{\prod }}\mathrm{SN}R_{i}=\overset{7}{\underset{i}{\prod }}\Gamma_{0}\frac{\pi {\left(\varepsilon +\delta \mathrm{\varepsilon cos}\omega_{s}t\right)}^{2}}{4\left[1+\exp\left(\frac{\varepsilon +\delta \mathrm{\varepsilon cos}\omega_{s}t}{k_{B}\left(T-\mathrm{\delta Tcos}\omega_{\mathrm{Ti}}t\right)}\right)\right]k_{B}^{2}{\left(T-\mathrm{\delta Tcos}\omega_{\mathrm{Ti}}t\right)}^{4}}$$

Where $\omega_{s}$ and $\omega_{\mathrm{Ti}}$ are the frequencies of the input energy and individual QD oscillations, respectively, and $\mathrm{SN}R_{i}$ is the SNR for tunneling through the *i*^*th*^ barrier. A QD absorbs signal energy and becomes active, oscillating either at the input frequency or its own natural frequency. The structured noise corresponds to intrinsic QD oscillations introduced into the system. Fitting to the data in Figure 7 (left) yields a main frequency $\omega_{s}=\;30000\mathrm{Hz}$ and a QD oscillation frequency $\omega_{\mathrm{Ti}}=3000\mathrm{Hz}$, meaning one QD in the string is active at 3000 Hz.

Similar behavior is observed in biology and neuroscience. For example, periodic heart contractions triggered by electrical stimulation are amplified and modulated *via* SR [34]. The envelope of the spike train shows clear periodic oscillations, with strength increasing alternately with input number – indicative of long-term potentiation arising from specific nonlinear coupling in cascaded neural strings at different stages. The envelope also reflects periodic biological signaling pathways to the heart. This work implies that memory can emerge in networks composed entirely of volatile basic units, suggesting that network-level memory is physically feasible [35].

**Figure 8.**
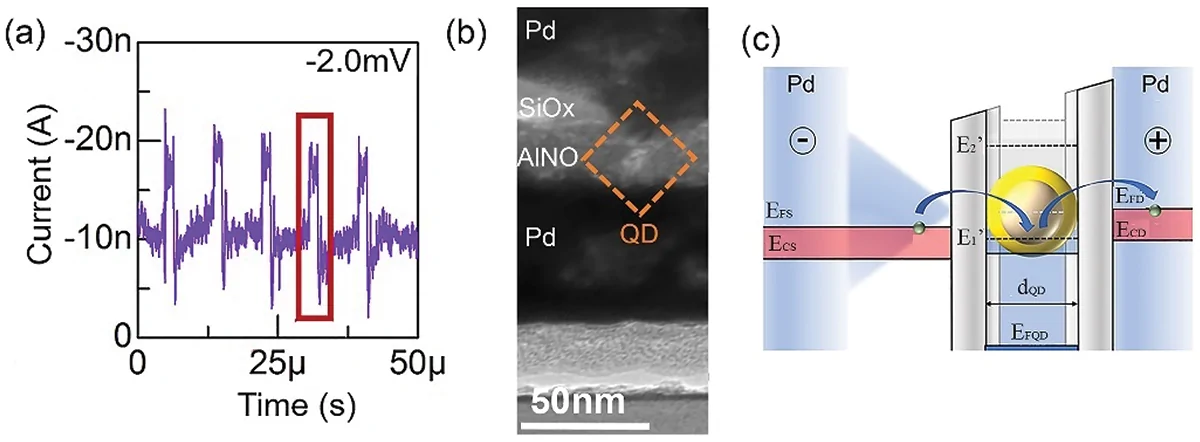
Quantized signal emitted by a phase-change quantum-dot (PCQD) [37] (a) Adaptive pulse generation depending on input level to the QD system. Responses to −1.8mV input. (b) Cross-sectional image for the state containing an individual c-NbO QD, which is about 3 nm circled in dashed orange box. (c) Schematic representations of structural variations and energy band model under negative bias. $E_{\mathrm{CS}}$ and $E_{\mathrm{FS}}$ indicate the conduction band and Fermi energy of source, respectively.$\;E_{\mathrm{CD}}$ and $E_{\mathrm{FD}}$ indicate the conduction band and Fermi energy of drain, respectively. $E_{F,\mathrm{NbO}}$ indicate the Fermi energy of NbO QD. $E_{1}$ and $E_{2}$ indicate the first and the second quantized energy in the potential well. (Adapted from Wan et al. [37] *Advanced Quantum Technologies* 2023, 6, 2300021. Reprinted with permission from Wiley).

It is widely accepted that signal emission and transmission are quantized at the nanoscale. The energy carried by a signal is proportional to the energy released by an elementary event in a nanosystem [36]. Precise control of signal magnitude and waveform enables high-precision computing and drastically reduces power consumption. Wan et al. successfully fabricated a NbO QD device in the middle of a Nb-doped AlNO film. The QD system emits periodic pulses (Figure 8(a)) when the input rises to a co-tunneling threshold or activates QD size oscillations [37]. The QD device has a double-barrier structure with the QD located in the quantum well (Figure 8(c)). An information–energy conversion model for this system confirms that the energy absorbed by the QD and released to form signals is quantized, comparable to the free energy of adding two complete atomic layers to the NbO QD. This work provides a more precise, low-power approach to neuromorphic computing by manipulating local activity and helps elucidate the spike-firing mechanism in NbO_x_ devices.

The material, size, and shape of QDs should significantly influence signal quantization in terms of frequency, strength, and computing unit in neural networks. The QDs in Figures 7 and 8 are cubic NbO phase. It is natural to ask whether NbO_2_ QDs can be obtained to realize similar signal quantization. Because its phase change relates to the variation of Nb alignment along the c-axis, the transition energy must be quantized according to the states with different Nb–Nb distances. However, it will be challenging to attain the stoichiometric ratio of NbO_2_ by precisely controlling oxygen pressure and reaction temperature during deposition. The shape of the amorphous phase usually approaches a sphere, but that of the crystalline phase varies depending on molecule (or atom) number. There should be a critical value for the reversible nucleation, which can be calculated using first-principles methods [38]. The quantized levels in the potential well should vary with the stacking size and shape of the crystalline QD. However, we are not able to find theoretical agreement for the amorphous QD.

VO_2_ resembles NbO_x_ as a type of Mott insulator that also deserves attention. Wei et al. quickly constructed a set of artificial voltage-control channels using VO_2_ based circuits after the NbO_x_ neuristor was reported [3,20]. This work has simulated almost all reported patterns of action potential. The VO_2_ device is also treated as a feasible I&F neuron in many artificial neural networks [8,39], exhibiting great potential in neuromorphic computing. Since the phase-change thresholds induced by both temperature and stress are very low, the VO_2_ device will be suitable for artificial sensory networks for robots.

## Resistive switching mechanisms of NbO_x_

To precisely control signal processing, one must understand and regulate the relationship between microstructural evolution and the RS process. As early as 1963, Geppert and Chopra discovered room-temperature NDR and oscillatory effects in niobium oxides [40,41]. Subsequent studies uncovered other intriguing physical phenomena [42], and extensive research eventually established that the NDR effect is closely associated with the NbO_2_ phase [43]. However, the dynamics of NbO_x_ are difficult to manipulate because they depend on the pre- and post-forming states and the applied signal waveform. I–V characteristics cannot be attributed to isolated factors, making it critical to analyze stable responses within defined signal-strength ranges. Advanced characterization techniques have gradually enabled in-situ and ex-situ investigations of these dynamic processes.

**Figure 9.**
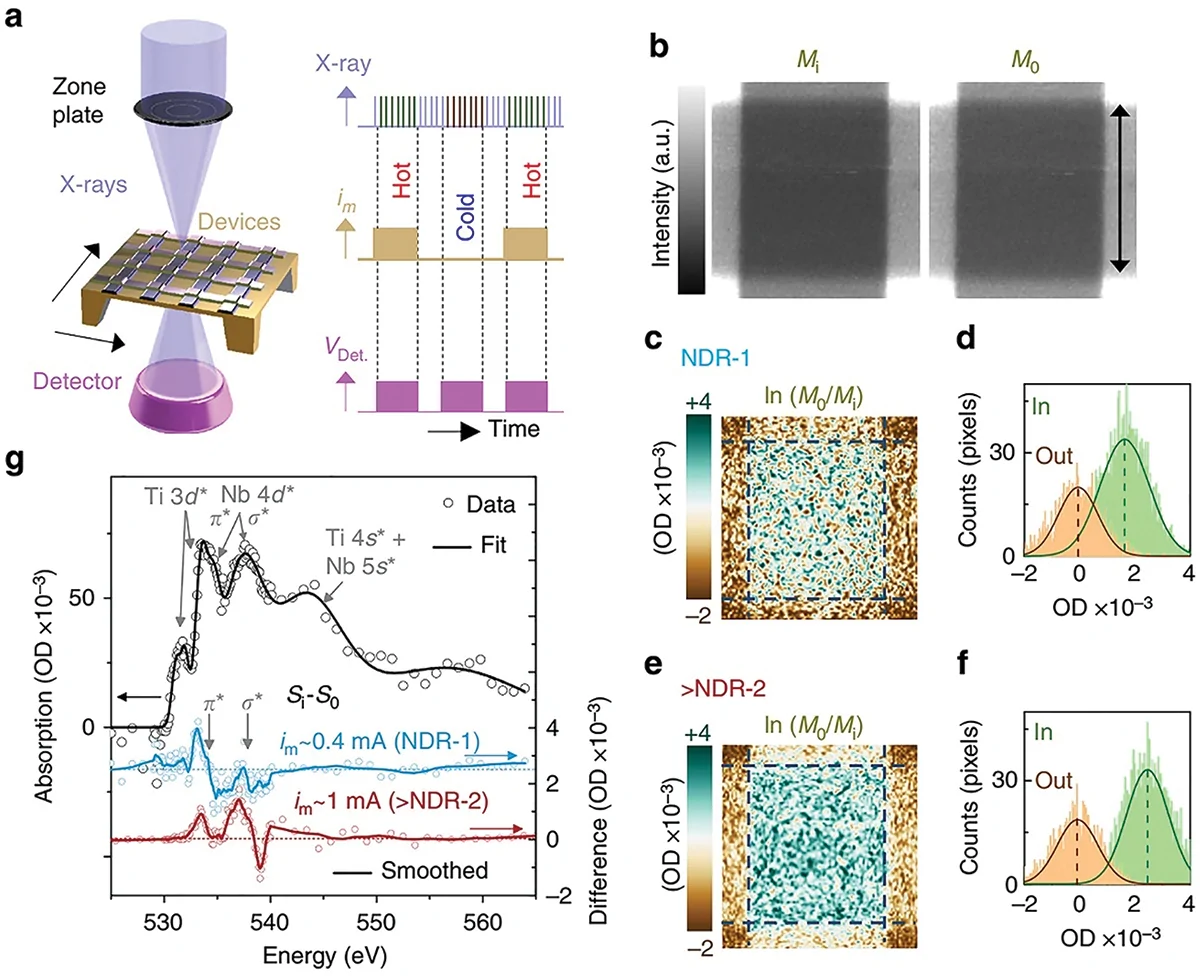
In operando synchrotron X-ray spectromicroscopy [44]. (a) Schematic of the experimental setup. (b) X-ray transmission maps of the crosspoint area of a fresh device with current (~0.4 mA, *M_i_*) and no current (M_0_) obtained using 533.2 eV X-rays. Scale bar is 2 μm. (c) Logarithmic ratio of the maps in (b). Intensity in optical density, OD. (d) Histograms of the data inside and outside the crosspoint area in (c). (e) Logarithmic ratio of maps similar to those in (b) obtained with a current sufficient exceed 1 mA and (f) its corresponding histograms. (g) X-ray absorption spectra of the material within the crosspoint. (Reproduced from Kumar et al. [44] under a Creative Commons Attribution 4.0 International License https://creativecommons.org/licenses/by/4.0/).

Figure 9 shows the evolution of local activity in NbO~x~ under a series of electrical stimuli, characterized by in-operando synchrotron X-ray spectromicroscopy [44]. The results confirm the occurrence of the Peierls transition in NbO_2_ (responsible for NDR‑2) and demonstrate that this current-driven transition occurs over a uniform region. A key open question is whether NDR-1 is related to the body-centered tetragonal (bct) phase. The bct phase is generally considered stable and unlikely to undergo amorphous transitions. Reversible crystal–amorphous transitions typically occur in systems with limited nucleation [38], where the supply of Nb or O atoms is restricted. Wan et al. showed that nanoscale NbO clusters reversibly form and dissolve in an AlNO matrix, inducing resistance fluctuations [45]. This observation confirms that NbO can act as a conductive phase in NbO_x_ films, consistent with low current densities at low temperatures. Transition between NbO and NbO_2_ near the NbO_2_ bct‑phase boundary may account for NDR‑1. This hypothesis is supported by surface-oxidation studies of Nb, which reveal sequential oxidation (NbO → NbO_2_ → Nb_2_O_5_) and the coexistence of NbO and NbO_2_ [46,47]. Nevertheless, further experimental evidence is needed to verify this mechanism in NbO~x~ thin films during signal processing. Moreover, while many studies have observed the NbO_2_ phase, its precise role in the NDR process requires careful investigation.

**Figure 10.**
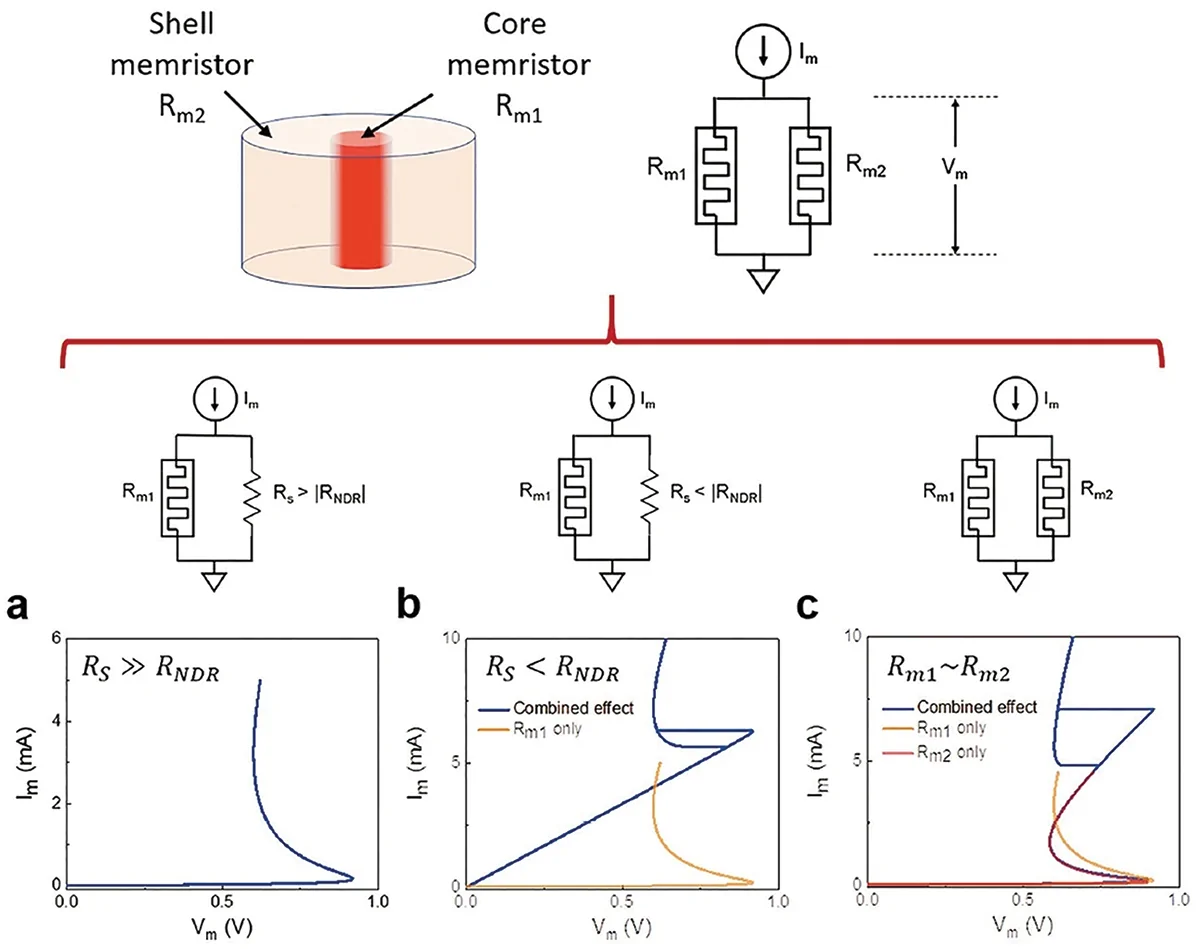
The core-shell model of local activity [49]. (a) The shell resistance is constant and greater than the core resistance. (b) The shell resistance is constant and lower than the core resistance. The orange line shows the I-V curve of *R*_m1_ without the parallel *R*_s_. (c) The shell resistance changes with current. The red and orange lines represent the individual I-V characteristics of *R*_m1_ and *R*_m2_, respectively. (Reproduced from Li et al. [49] *Advanced Functional Materials* 2019, 29, 1905060. Reprinted with permission from Wiley).

Since NDR phenomena in NbO_x_ also appear in other oxide systems [48], a general core–shell model has been proposed to describe current redistribution between a core and a shell, reproducing characteristic I–V curves [49]. Local activity is assumed non-uniform along the radial direction, characterized by a resistance gradient that can be positive or negative depending on fabrication, forming, and electrical-stress conditions (Figure 10). When the shell resistance $R_{m2}$ is treated as a fixed series resistor $R_{s}$, a high $R_{s}$ yields an S‑shaped I–V curve, while a low $R_{s}$ produces a snapback curve. If both $R_{m1}$ (core) and $R_{m1}$ (shell) are variable, the I–V curve takes a combined S‑/snapback shape (Figure 10(c)). This model explains abrupt current redistribution during RS and accounts for several typical I–V curves in both volatile and nonvolatile oxide systems [48]. However, more accurate dynamic models are required that incorporate instantaneous variations in material structure and physical parameters related to local activity. Phase‑change or volatile RS materials possess self‑feedback and structural‑recovery capabilities and may not require core–shell coupling. Yu et al [16] and Kumar et al. [7] independently developed third‑order chaotic dynamical systems without shell resistors to accurately simulate threshold‑driven spike trains. A common limitation, however, is that the first NDR effect in S‑shaped curves remains incompletely explained. Furthermore, unexplained physical mechanisms likely exist in NbO_x_ materials, as evidenced by ultra‑high‑frequency (~100 MHz) ‘parasitic oscillations’ that cannot be accounted for by current theories [41].

**Figure 11.**
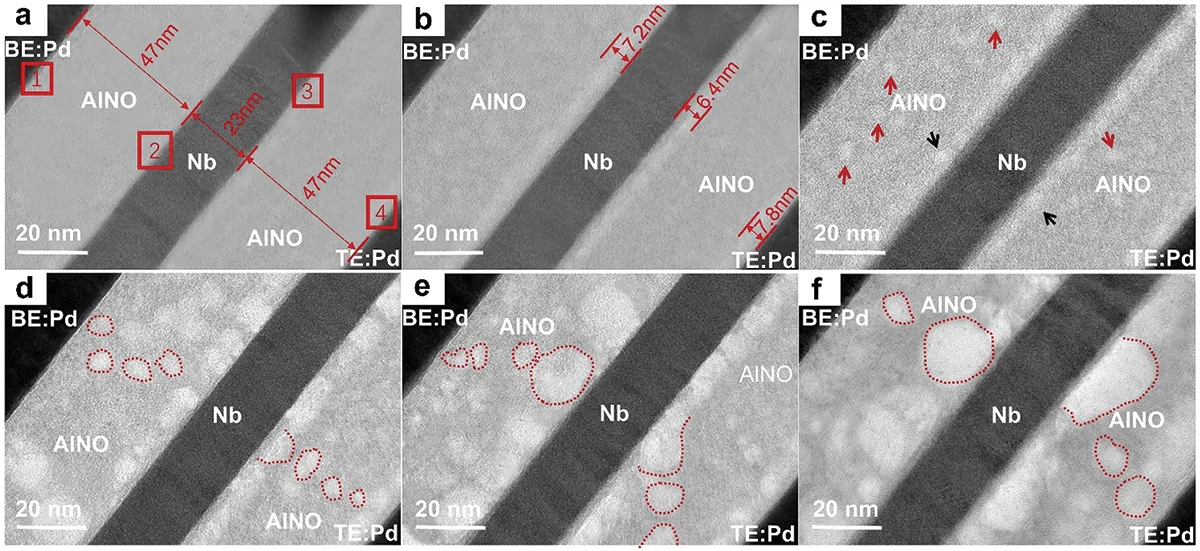
Reversible NbO nano-clusters appear in AlNO layers. The input signal series control the statistic size and distribution [45]. ex-situ high revolution transmission electrical microscopy examinations on (a) the initial state, the States after 100 voltage sweeps with sequence of (b) 0→−0.5→0→0.5→0V, (c) 0→−1→0→1→0V, (d) 0→−2→0→2→0V, (e) 0→−3→0→3→0V, and (f) 0→−4→0→4→0V. BE, bottom electrode; TE, top electrode. (Reproduced from Wan et al. [45] *Small* 2022, 18, 2105070. Reprinted with permission from Wiley).

An important phase governing RS in NbO_x_ thin films is NbO. Early surface‑oxidation studies of Nb found the co-existence NbO, NbO_2_ and Nb_2_O_5_ when the oxidation is very thin [46,50]. We hypothesize that NbO clusters form within oxygen-deficient NbO_x_ films (2 < x < 2.5). First-principles calculations and experiments confirm that NbO is conductive, similar to NbN [51]. Many studies use transition-metal nitrides as electrodes; nitrogen atoms may diffuse into NbO_x_ films during forming and electrical manipulation, further influencing switching mechanisms. NbO commonly appears along conductive filaments because oxygen vacancies are readily driven into these locally active regions. However, systematic studies are lacking due to the difficulty of isolating and characterizing the NbO phase. Figure 11 illustrates the formation of NbO nanoclusters: under an applied electric field, Nb atoms lose electrons and migrate into AlNO films. With limited Nb and O availability, nanoscale NbO clusters undergo reversible nucleation without coalescence [38]. Under these conditions, device responses to electrical stimulation exhibit both synaptic plasticity and neural spike firing, resembling high-order memory phenomena recently reported in neuroscience [52]. Thus, constructing nanoscale systems that emulate multi-ionic channels in neural membranes holds great promise for discovering new neuromorphic computing paradigms.

## Conclusions

**Figure 12.**
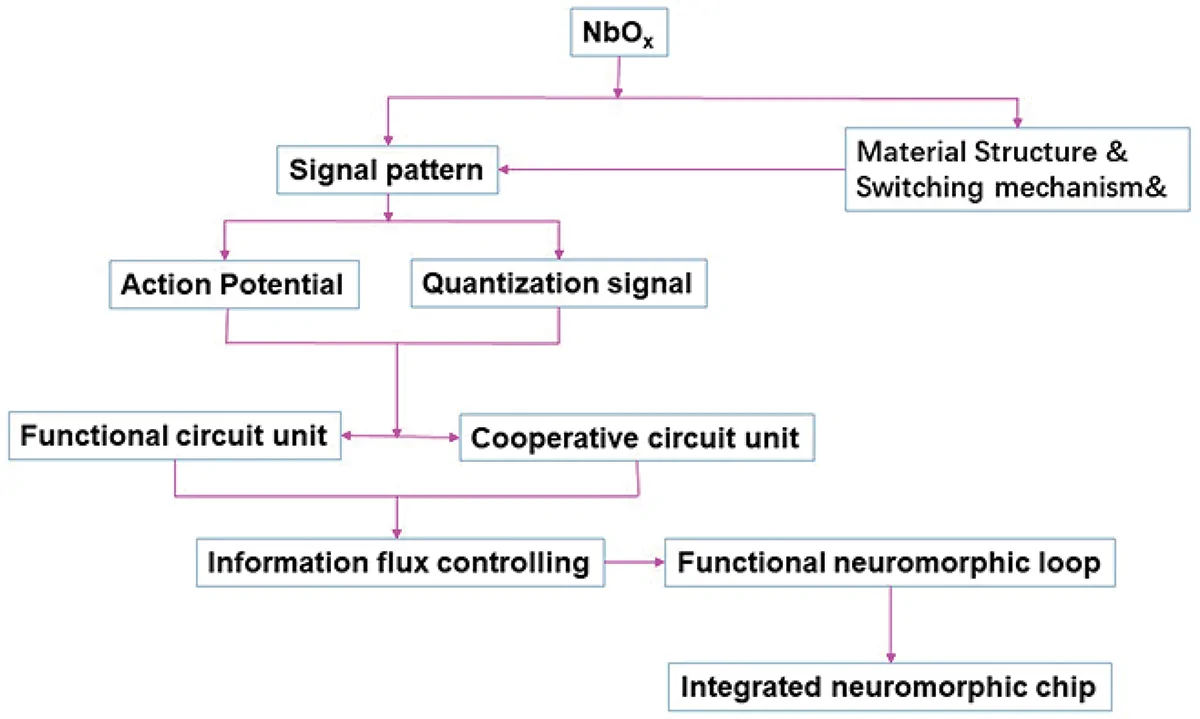
Study of signal processing and resistive switching of NbO_x_ aims to fabricate novel neuromorphic chip comparable to biological neural network.

Niobium oxide materials and devices play a vital role as neuron elements in artificial neural networks. We highlight their exceptional potential for advanced signal processing based on two key insights. First, niobium oxides exhibit rich high-order chaotic dynamics governed by their phase composition, enabling diverse information-processing tasks at ultra-low power. Second, despite their simple crystalline structures, their microstructures can be adaptively modulated, facilitating the construction of complex signal-processing systems. This paper reviews the fundamental signal-processing models and resistive switching mechanisms of NbO_x_. These two areas demand extensive and in-depth research from physicists and engineers, opening broad opportunities for innovation in the development of highly biomimetic artificial neural networks, as suggested in Figure 12. Constructing bionic circuits and neural networks using NbO_x_ materials and devices remains a great challenge. Every process in the summary figure has commercial potential. These goals deserve the cooperation and endeavors of scientists and engineers.

## Methods

For electrical measurements in Figure 2, the DC test was performed on an Agilent B1500A semiconductor parameter analyzer, the pulse tests were finished by using Keysight 81160 A pulse generator and a Keysight Infinii Vision MSO-X 3104 T oscilloscope. Electrical characterizations in Figures 3, 4, 7, 8, 11 were performed using an Agilent B1500A semiconductor parameter analyzer with a B1530 waveform function generator from Keysight Technologies. The electrical measurements of Figure 10 were performed using an Agilent B1500A semiconductor parameter analyzer.
